# Porous Silica Nanomaterials as Carriers of Biologically Active Natural Polyphenols: Effect of Structure and Surface Modification

**DOI:** 10.3390/pharmaceutics16081004

**Published:** 2024-07-29

**Authors:** Ivalina Trendafilova, Margarita Popova

**Affiliations:** Institute of Organic Chemistry with Centre of Phytochemistry, Bulgarian Academy of Sciences, BG-1000 Sofia, Bulgaria; margarita.popova@orgchm.bas.bg

**Keywords:** porous silica, drug delivery, surface modification, natural bioactive compounds, polyphenols

## Abstract

For centuries, humans have relied on natural products to prevent and treat numerous health issues. However, biologically active compounds from natural sources, such as polyphenols, face considerable challenges, due to their low solubility, rapid metabolism, and instability, which hinder their effectiveness. Advances in the nanotechnologies have provided solutions to overcoming these problems through the use of porous silica materials as polyphenol carriers. These materials possess unique properties, such as a high specific surface area, adjustable particle and pore sizes, and a surface that can be easily and selectively modified, which favor their application in delivery systems of polyphenols. In this review, we summarize and discuss findings on how the pore and particle size, structure, and surface modification of silica materials influence the preparation of efficient delivery systems for biologically active polyphenols from natural origins. The available data demonstrate how parameters such as adsorption capacity, release and antioxidant properties, bioavailability, solubility, stability, etc., of the studied delivery systems could be affected by the structural and chemical characteristics of the porous silica carriers. Results in the literature confirm that by regulating the structure and selecting the appropriate surface modifications, the health benefits of the loaded bioactive molecules can be significantly improved.

## 1. Introduction

Nanotechnology is considered one of the most rapidly developing sciences of the 21st century due to its economic potential in the production of new or optimized products and its expected contributions to minimizing ecological stress and resource consumption [1]. Nanotechnology profoundly influences many areas of human life. Its proper application can lead to remarkable advancements in medicine, industry, electronics, and other vital sectors.

Nanoparticles with a size <500 nm have been intensively studied in recent decades as carriers in drug delivery systems (DDS) due to their ability to optimize the physicochemical and pharmacokinetic properties of biologically active compounds (BAC) loaded onto them. Nanomaterials have the ability to reduce the toxicity, ensure controlled release, and to achieve targeted delivery of bioactive molecules from natural and synthetic origins [2,3]. Moreover, the physicochemical properties of the materials used as carriers can be easily controlled by changing their composition (organic, inorganic, and hybrid), size, shape (spherical, rod-like) and surface characteristics (surface charge, modification with functional groups, coating with polymers, attachment of directing groups-enzymes, antibodies, etc.) [4].

Many challenges associated with drug delivery efficiency can be overcome by the use of nanoscale carriers: (i) due to their small size, the particles have the ability to cross physiological and biological barriers and can even penetrate fine capillaries and enter the cell, allowing efficient accumulation of BAC at target sites [5,6]; (ii) prolonged drug release at the target site can be achieved, over a period of days to weeks, to reach the desired therapeutic concentration for the time required for optimal treatment efficacy [7]; (iii) the surface of the particles can be designed to alter their biodistribution, directing them to the target organ or tissue [8], reducing the off-target side effects; and (iv) increasing the solubility and the stability of BAC in the biological environment, leading to higher bioavailability and prolonged half-life of the BAC in the bloodstream and avoiding its premature loss due to rapid metabolism.

Porous silica nanomaterials provide numerous benefits due to their unique structures, and extensive research has been conducted on their synthesis methods and possible applications across various fields. As defined by IUPAC, porous materials are categorized into three classes: microporous (pore size < 2 nm), mesoporous (2–50 nm), and macroporous (>50 nm) materials [9]. Additionally, the term “nanoporous” is becoming increasingly common. Within the family of microporous materials, zeolites are the most well-known. They are characterized by narrow and uniform micropore size distribution due to their crystallographically defined pore system. However, zeolites face limitations when large molecules are involved, due to restricted mass transfer within the micropores. Efforts to improve the diffusion in the pores have focused on increasing zeolite pore sizes, reducing zeolite crystal sizes, or incorporating an additional mesopore system within the microporous crystals [10,11,12]. Recent advances in surface functionalization and control of morphology of mesoporous silicate materials make them extremely attractive for a wide range of applications in the field of nanomedicine. Their highly developed surface and pore volume are a prerequisite for loading a significant amount of biologically active substances.

## 2. Porous Silica Nanomaterials 

**Figure 1:**
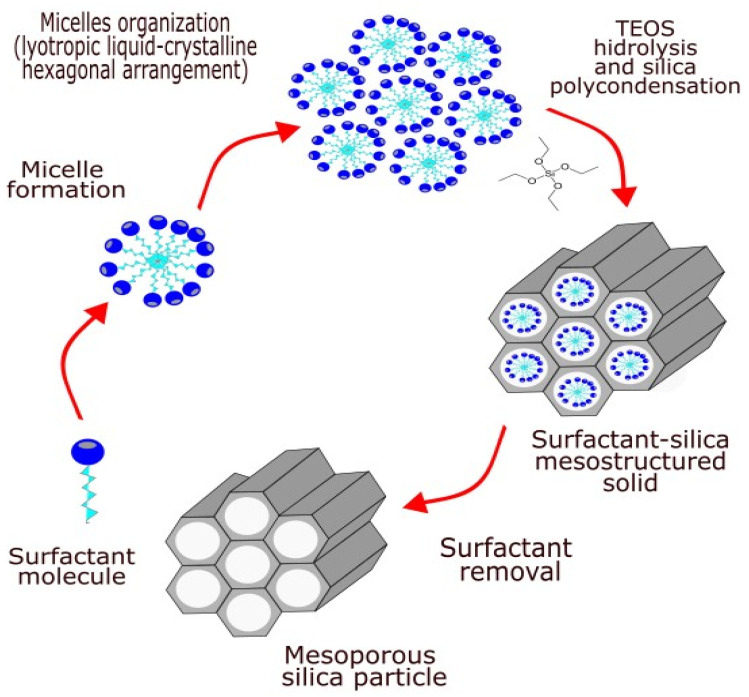
General concept for synthesis of mesoporous silica from micelle template. Reproduced with permission from [18], Elsevier, 2020.

The first synthesis of an ordered mesoporous silica material was described in the literature in 1970 [13]. This material differs from the zeolites by its chemical composition, as in this case the material is not an aluminosilicate containing anionic Si-O-Al linkages, but a silicate containing anions consisting only of silicon and oxygen. However, the remarkable features of this product were not recognized because of its insufficient characterization [14,15]. In 1992, scientists at Mobil Oil Corporation developed a similar material and discovered its remarkable properties, thereby opening up an entirely new field of research in silica-based materials [16]. The general concept of mesoporous silica synthesis is presented in Figure 1. Mesoporous silicate materials are synthesized based on the liquid-crystal template mechanism, in which long-chain surfactants are used as structure-determining agents [17]. 

The obtaining of mesoporous materials includes three main steps: synthesis, drying, and removal of the template. In the synthesis step, several crucial stages are identified: hydrolysis of the organosilane, seeds formation, and aging (polycondensation). The most frequently used sources of silicon are organosilanes. The templates used are usually self-organized surfactant molecules. The first structure-directing agents mentioned in the literature are quaternary ammonium salts, which at a certain concentration (critical micelle concentration) in solution are self-organizing into structures around which the silicate structure is formed. The possible types of interactions between the organic templates and silica precursor are determined by the charge of the template (surfactant) (S^+^ or S^−^), the inorganic phase (I^+^ or I^−^), and the presence of intermediate ions (X^−^ = Cl, Br, OH or M^+^ = Na, K). Therefore, several interactions involved into the mechanisms for silica-template mesophase formation are possible: electrostatic S^+^I^−^, S^−^I^+^, S^+^X^−^I^+^, S^−^M^+^I^−^; neutral (hydrogen bond) S°I°, N°I° and covalent S-I.

In S°I°, N°I° mechanisms, neutral surfactants such as primary amines and polyethylene oxide are used as structure-directing agents, allowing the formation of silicas with ordered hexagonal arrangement of the pores and a disordered structure with a “wormhole-like” pore channels, respectively. The silica materials obtained by the S^−^I^+^ or S^−^M^+^I^−^ mechanisms, where the templates are anionic, usually have a layered and with disordered structure.

The mechanism used by Mobil is S^+^I^−^ and pH = 9–14, where the inorganic precursors are negatively charged. It has been found that the low pH values provide priority for the self-organization of silicon precursor molecules due to electrostatic forces and hydrogen bonds, while in acidic conditions the silicon source is easily protonated and positively charged (S^+^X^−^I^+^). Therefore, synthesis under acidic conditions has the advantage of proceeding rapidly and at low concentrations of template compared to synthesis under alkaline conditions and high temperature [19]. The pore size of the materials can be easily controlled by changing the length of the alkyl chain of the surfactant used or by adding swelling agents (organic molecules). For example, in quaternary ammonium salts of C_n_TMABr type, where n = 8, 10, 12, 14, 16, 18, the pore size increases by 0.225 nm with each increase by one C-atom in the template chain.

A predominant part of the materials from the M41S group (Mobil Composition of Matter No. 41) were obtained by synthesis in a basic medium, where quaternary ammonium salts (C_n_H_2n+1_) (C_m_H_2m+1_)_3_NX (n = 6–22, m = 1–4, X = OH/Cl, OH, Cl, Br or HSO_4_) were used as templates. This is an S^+^I^−^ mechanism using a cationic template and negatively charged silicon precursors (pH=9–14). In the preparation of some mesoporous materials (MCM-41 and MCM-50), sulfates, sulfonates, phosphates, and carboxylic acids with long alkyl chains were used as templates. The first and the most thoroughly studied member of the M41S family is the mesoporous silicate type MCM-41. It is characterized by a highly ordered hexagonal array of unidimensional cylindrical pores with a diameter of 1.5–3.0 nm, high specific surface area (up to 1200 m^2^g^−1^), and large pore volume. However, the walls closely resemble amorphous silica. The variation of the synthesis conditions and the ratio of reagents results in the development of new and different types of structures [20].

For the neutral synthesis mechanism (S°I°) of mesoporous silicates, nonionic templates are used. The synthesis is carried out in an acidic media, where non-ionic triblock copolymers (EO_x_PO_y_EO_x_) with large polyethylene oxide (EO)_n_ and polypropylene oxide (PO)_m_ chains are used as structure-directing agents. In this case, the cationic silica particles are organized around the ethylene oxide units, forming a mesoporous structure-type SBA. The ordered mesoporous silicas of SBA type were first prepared at the University of Santa Barbara on the basis of the neutral mechanism [21]. Unlike M41S-silicates, SBA materials have larger mesopores, although in terms of pore topology they are structural analogues of MCM materials. The walls of the SBA are 3–6 nm thick, which makes them more stable in conditions close to hydrothermal; in comparison, the wall of materials prepared in the presence of anionic template such as MCM-41, MCM-48 are thinner ~ 1.5 nm. Silica materials of type SBA-15 are of great interest among the mesoporous silicas prepared by neutral mechanism. They are porous materials with two-dimensional hexagonally arranged mesopores and micropores localized in the walls and connecting the mesoporous channel structure. The average diameter of mesopores can vary in a wide range of 4.6–30 nm, and of micropores, from 0.5 to 3 nm. It has been found that the particles and pores of the resulting SBA-15 materials can be controlled by changing the synthesis conditions. The micropores in the walls of SBA-15 materials are formed by the polyethylene oxide (PEO) residues of the triblock copolymers present in the aqueous solution, while the polypropylene oxide moieties (PPO) are more hydrophobic and form the internal structure of the mesopore. As a result of the change in the length of the PEO fragments of the copolymer, materials with different amounts of micropores and wall thicknesses are obtained. On the other hand, varying the length of the PPO parts will lead to a difference in the diameter of the mesopores. Moreover, the change in synthesis conditions such as temperature, stirring rate, pH, and addition of electrolytes and salts allows control of pore size and thus largely provides the ability to regulate the basic textural and morphological characteristics of SBA-15 [22].

The removal of the organic template from the obtained organic–inorganic composite is the final step of the porous materials’ preparation. This process affects the structure of the material and the distribution of the pores. One of the most commonly used approaches is the thermal decomposition of the organic template (calcination). This procedure leads to the complete removal of the organic part, but very often causes shrinkage of the pores. An alternative method of removing the template is by extraction with acid, neutral salt solution, alcohol, ammonium acetate, and others. A method which is also used involves removing the part of the template contained in the mesopores by extraction and subsequently decomposing the organic residues that have penetrated the pore walls by thermal destruction. In this way, materials with significantly higher meso- and microporosity are obtained. In the literature can be found information for some less conventional approaches for template removal, such as supercritical CO_2_ extraction, ozone or ionic liquid treatment, microwave- or ultrasonic-assisted treatment, and plasma technology [23].

## 3. Biomedical Application of Porous Nanomaterials

**Figure 2:**
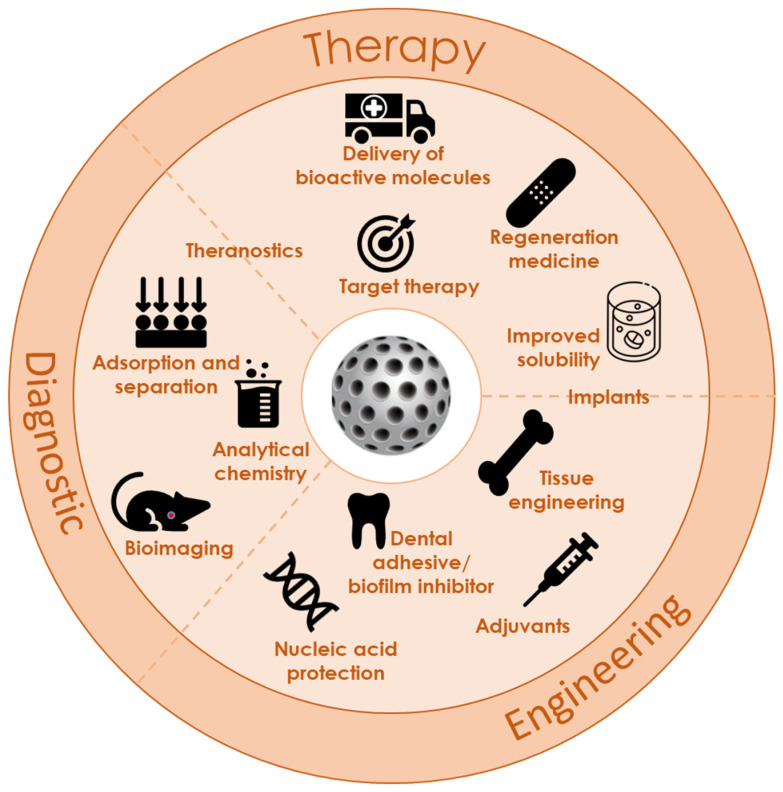
Application of mesoporous materials in various biomedical fields.

Mesoporous silica nanoparticles are highly attractive for various biomedical applications due to their high specific surface area, large pore volume, uniform porosity, stable aqueous dispersion, excellent biocompatibility, in vivo biodegradability, and ability to be functionalized with various organic groups and/or metal (oxide) particles. These properties make them ideal for controlled drug and gene delivery, bone tissue regeneration, cell tracking, protein or enzyme immobilization, and more (Figure 2). In addition, silica nanoparticles are generally recognized as safe and listed as an anticaking agent by the United States’ Food and Drugs Agency and as food additive (E551) in the European Union [15,24,25,26].

In 2001, Vallet-Regi et al. published research work that proposed for the first time mesoporous silica materials as carriers for drug delivery [10]. They were applied as drug carriers in delivery systems for controlled drug release in order to achieve prolonged and more precise drug administration. Mesoporous materials ensure the homogeneous distribution of bioactive molecules throughout the matrix, unlike conventional polymeric materials [27,28,29,30]. Several key factors should be considered when designing delivery systems based on porous silica: (i) the size selectivity—the pore size of the mesoporous materials determines the size of the molecule that can be hosted into the mesopores; (ii) interactions—the chemical and the electrostatic interactions between bioactive molecules and the mesopore wall can be easily modified in order to increase the affinity of the host molecule to the carrier and to modify their release profiles; (iii) administration—depending of the way of application the size, shape, and surface chemistry of the silica, particles can be modified to enhance the efficiency of the treatment. Due to their unique pore structure, when silica materials are applied as carriers in delivery systems they can solve the problem of low water solubility, and respectively the bioavailability of some bioactive molecules, by finely dispersing them in the pores of the carrier, preventing formation of bigger, hardly soluble crystals of the pure drug. Another major problem that can be solved by using porous silica particles as delivery vehicles is reducing the undesirable side effects of some therapeutics by encapsulating them and thus assuring targeted delivery and controlled release.

For many years, porous silica and zeolites have been used to design highly efficient systems for delivery of synthetic therapeutics commonly used in medical practice. However, the latest tendencies in therapies and healthy lifestyles are mainly focused on bioactive compounds from natural origin. The botanical and plant-derived drugs market has increasingly grown worldwide and the estimated growth for the next 5 years is 8.18% [31]. Encouraged by governmental initiatives and private funds, many academic and industrial groups focus their research on the development of formulations based on naturally derived health-promoting compounds. 

## 4. Polyphenols and Their Biopharmaceutical Properties

**Figure 3:**
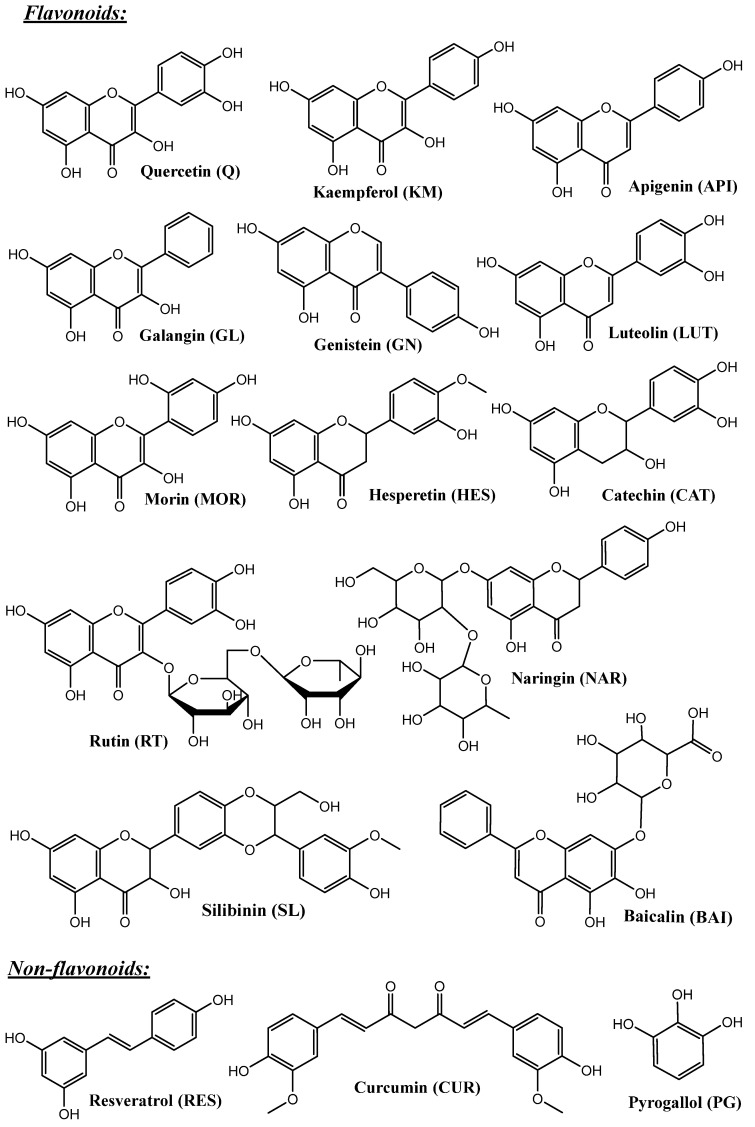

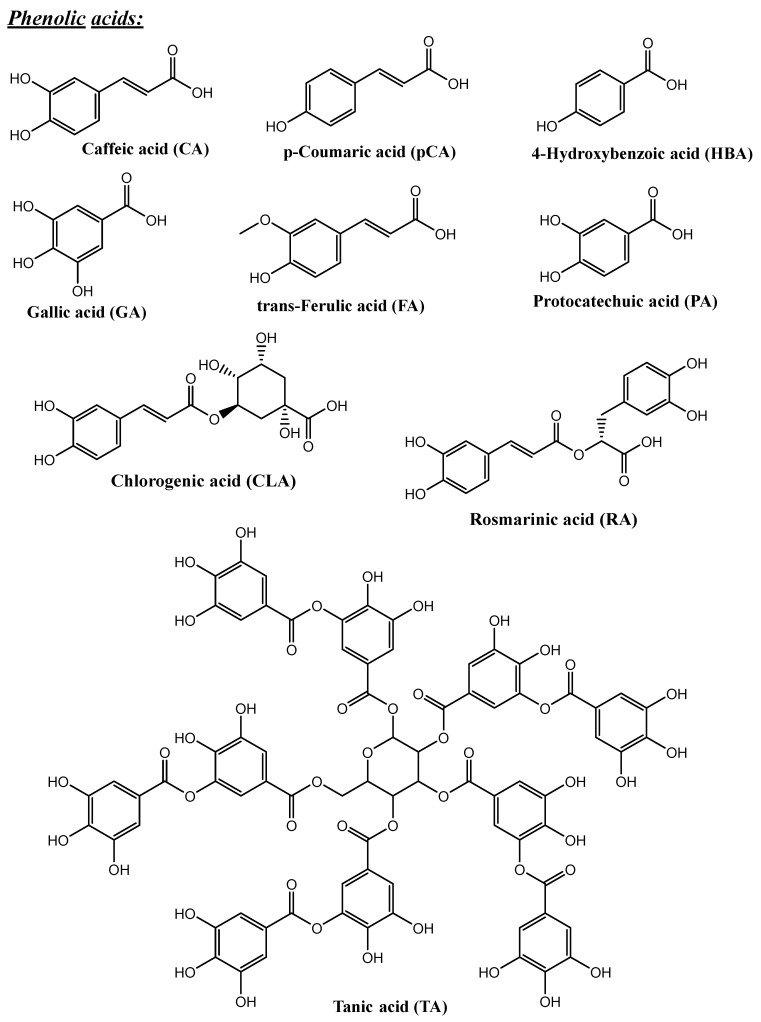
Chemical structure of some polyphenols: flavonoids, non-flavonoids, and phenolic acids.

One of the biggest groups of natural bioactive compounds considered extremely promising for prevention and treatment of numerous health issues is the group of polyphenols (Figure 3). According their structure, they can be divided in the following groups: phenolic acids, flavonoids, non-flavonoids (stilbenes), phenolic alcohols. The phenolic acids are presented by hydroxycinnamic and hydroxybenzoic acids with C_1_–C_6_ and C_3_–C_6_ structures, respectively. Flavonoids can be additionally separated in sub-classes: flavanones, flavanols, flavonols, isofalvons, flavons, and anthocyanidins, on the basis of the oxidation state of the central carbon. Agents from this group are found in fruits (apples, citruses, berries, etc.) and vegetables (red onion, broccoli, etc.), cereals, olives, extra virgin olive oil, red wine, coffee, green and black tea, chocolate, and flowers, and they are the most important antioxidants in our food [32,33,34].

Many studies prove their free radical scavenging capacity, antioxidant, anti-inflammatory, analgesic, anti-anxiety, anti-tumor, anti-allergic, antibacterial, antifungal, antiviral, and antidiabetic activity [35,36,37,38]. Despite the proven high biological activity and health benefits, the development of an efficient pharmaceutical formulation for the application of a compound from natural origins meets challenges related to the low water solubility of most of these compounds, which is an obstacle in achieving optimal bioaccessibility. Other major obstacles to reaching their pharmacological potential are burst release, rapid metabolism, and fast excretion of the dietary polyphenols after administration, which leads to low bioavailability [39]. An effective way to address these challenges and achieve high therapeutic efficiency with polyphenols is to develop a suitable porous silica-based delivery system for their application. 

## 5. Porous Silica Materials with Different Structures as Carriers in Delivery Systems of Polyphenols

Porous silica can be obtained with a variety of shapes (spherical, tube, cubic, etc.), sizes (50 nm–5 µm), surface morphologies, pore structures, and volumes. All of these characteristics can significantly influence the properties of the material. Many studies are focused on clarifying the relationship structure-properties. For example, spherical MCM-41 with hexagonal arrangement of 2D cylindrical mesopores, KIL-2 with a wormhole pore structure, and zeolite BEA with a 3D nanosized pore system have been used as carriers of natural polyphenol resveratrol (RES, Figure 3). This compound is extremely photosensitive and with low chemical stability, where the trans-form is with higher biological activity, which limits its beneficial therapeutic effects. Thus, loading of resveratrol in nanoporous silica systems is a premise for the stabilization of the most active form and protection from photoinduced degradation. The resveratrol was loaded in these three carriers by a solid-state procedure that ensures simultaneous amorphization and loading of the poorly water-soluble drug. The results for resveratrol loading were 32% for MCM-41, 40% for BEA, and 37% for KIL-2. The obtained numbers for percentage-loaded resveratrol indicate that the structure of the material plays a role in the loading capacity. The release of resveratrol in phosphate buffer (pH 7.4) was repeated three times over one-week intervals, and the results revealed the presence only of trans-resveratrol. This indicates that loading resveratrol onto nanoporous carriers prevents its transformation from the bioactive trans-form to the less active cis-form. Consequently, loading resveratrol on powdered mesoporous silica carriers stabilizes the compound and enhances its solubility.

The release profiles of the resveratrol from the loaded supports are similar and total release in about three hours was achieved for all resveratrol-loaded samples. On the contrary, the free resveratrol dissolves much slower. For the KIL-2 support slower initial release profiles are observed and that can be explained by the presence of much larger mesopores formed among the nanoparticles of the KIL-2 carrier, where the formation of bigger hardly soluble crystals is possible. The internal and external mesoporosity of MCM-41 and both micro- and mesopores of zeolite BEA offers a possibility of selective access for resveratrol in and out of the pore systems of these supports. This can lead to variations in observed release kinetics. For example, the narrower pore channels of the zeolite carrier showed a slower resveratrol release rate compared to the same of MCM-41 [40].

Another study compares the mesoporous silica type MCM-41(2D pore structure; spherical particles with sizes in the range of 150–400 nm) and MCM-48 (3D pore structure consists of the interlaced pore system divided by a continuous pore wall; quasi-spherical particles with sizes in the range of 150–400 nm) as carriers for caffeic acid (CA), p-coumaric acid (p-CA), and trans-ferulic acid (FA). The results from thermogravimetrical and surface area measurements evidenced higher loading of all three polyphenols on the MCM-41 carrier in comparison with the MCM-48. This observation can be explained by the difference in the pore structure of the used materials where, despite the higher pore volume of MCM-48, higher loading of MCM-41 can be a consequence of the easier access into the cylindrical pores. For the same reason, the release profiles of polyphenols from MCM-41 and MCM-48 supports differ. For the samples loaded with caffeic acid, a significant difference can be observed for both silica supports—the release from MCM-48 is slower and the degree of CA recovery for the studied period is only ~50%, while for MCM-41/CA it was ~70%. A similar tendency was noted for FA-loaded samples. Unlike those of CA and t-FA, the release profiles of p-CA for both MCM-41 and MCM-48 were very similar. This indicates that the chemical structure of the loaded molecules and their interactions with the support are also important factors that can influence the release properties of the obtained systems [41].

A detailed study compared the two mesostructured silicas with wormhole-like pore arrangement (quasi-spherical HMS (1.4–5.2 µm) and spherical MSU-2 (0.5–1.9 µm) particles) with hexagonal mesostructured silica (rod-like SBA-15 particles (1 × 0.5 µm)) as carriers of quercetin (Q) and naringin (NAR). Their adsorption capacity and encapsulation efficiency, as well as their ability to modify the release kinetics and antioxidant activity of the loaded polyphenols, were evaluated. For quercetin, HMS showed higher encapsulation and loading capacity than SBA-15 and MSU-2, whereas for naringin the loading capacity of the three supports was similar. The study showed that the HMS material required more time (2 h) than SBA-15 (30 min) to achieve maximum adsorption capacity of quercetin, but it can be considered as more suitable due to its higher encapsulation efficiency. For the naringin, all carriers achieved maximum encapsulation efficiency in 30 min. HMS showed not only the greatest quercetin encapsulation but also the highest amount released at tested pH values (2.0 and 7.0). These observations could be explained by the molecular size of these polyphenols and the different textural properties of the materials, and in particular with the pore size, which increased from 25.2 Å, through 31.2 Å to 55.5 Å for HMS, MSU-2, and SBA-15, respectively [42]. 

**Figure 4:**
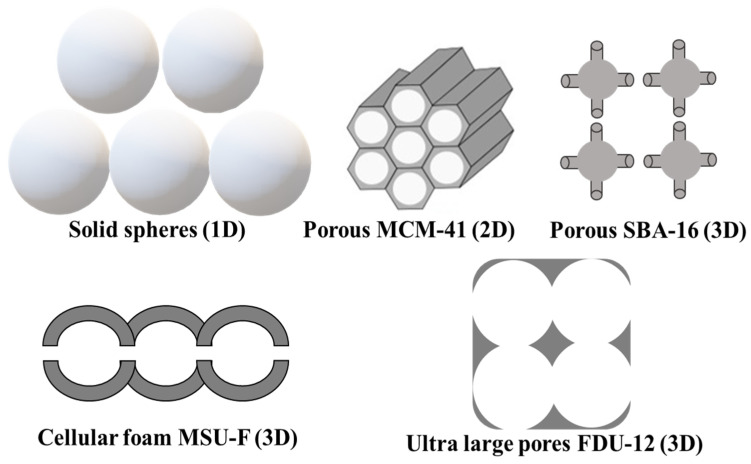
Schematic representation of 1D, 2D, and 3D pore structure of different silica materials.

The significance of the pore structure in the efficiency of the delivery system is well described in a comparative study that used one-, two-, and three-dimensional silica nanocarriers (Figure 4, GA was used as a model drug). For the studied carriers, the absorption of GA decreased in the following order: SBA-16 (3D)∼KIT-6 (3D) > MCM-41 (2D) > ultra large pore FDU-12 (ULPFDU-12; 3D) > Q10 (1D)∼mesostructured cellular silica foam (MSU-F). The analysis showed that the 3D-type silicas accommodated GA in amorphous (non-crystalline) form, while for Q10 silica, ULPFDU-12, and MSU-F the GA was found only in crystalline form. This way, using the same impregnation procedure and conditions, it is possible to control the crystallinity of the loaded compound only by changing the structure of the porous silica. On the other hand, the most favorable impregnation of GA occurred at pH 3, where the tick walls of the 3D material showed significant stability. In contrast, ULPFDU-12 and MSU-F supports suffer structural damage and textural changes during the impregnation procedure in acidic conditions [43].

Regarding the pore size:loaded molecule size ratio, controversial opinions can be found in the literature, and no particular pattern is observed. For example, for the GA (0.8 nm) it was found that an increase of the MCM-41 pore size from 2.4 to 3.4 nm enhanced the loaded amount two times [44], but it was also observed that the adsorption of GA onto SBA-15 (pore size 6 nm) was lower than the adsorption on MCM-41 (pore size 2.5 nm) [45]. On the basis of these results, it can be assumed that there is an optimal ratio pore size:molecule, but no information in the literature was found. 

These results clearly show that the pore structure, textural properties, and morphology of the mesoporous support play a crucial role in the adsorption capacity and release profiles and can influence the properties of the obtained delivery system. Thus, when the delivery system is designed, the choice of porous silica support type is essential and has to be made in accordance with the requirements for application of a particular biologically active substance (Table 1).

## 6. Functionalized Porous Silica Materials as Carriers in Delivery Systems of Polyphenols 

The surface of silica materials is mainly composed of OH-groups, which are not highly selective in reactions of adsorption. A suitable approach to increasing the selectivity of the adsorbent or affinity of the target molecules to the adsorbent is functionalization of the surface with appropriate moieties. In this case, the presence of OH-groups on the silica surface is an advantage, as these groups allow easy introduction of different organic groups in one-step procedures. The surface OH-groups can also play a role in the coordination of some metal ions. The high specific surface area and the presence of pores and surface defects make silica materials easy to modify with metal or metal oxide nanoparticles. In the literature, two main approaches for modification of the silica surface are described: (i) in sito during the synthesis of the silica material; and (ii) post-synthesis modification. In the first approach, the modifying agent is directly added with the silica precursor during the silica formation reaction. In this case, the modifying agent is more likely to interact with the silica species during the condensation reaction and to be strongly immobilized into the silica framework. The disadvantages of this approach are disruption of the structural order of the final product (more defects in the structure) and loss of reachable active sites from the modifying agent due to its incorporation deep into the silica walls. The post-synthesis approach for the modification of the porous silica materials’ surface is often preferred because of the preservation of the pore structure of the parent silicate during the functionalization procedure and the possibility of better control of the amount of immobilized specific groups. The post-synthesis modification can be realized by different techniques, such as incipient wetness impregnation, modification in solution, template-ion exchange, dry mixing, etc. [68].

### 6.1. Functionalization with Organic Groups

**Figure 5:**
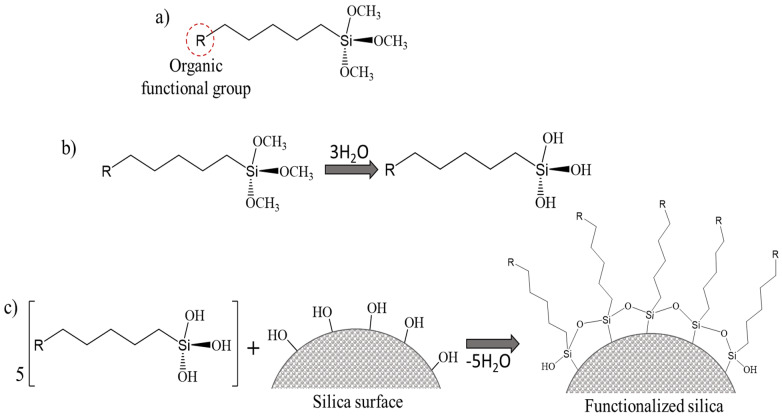
Structure of organosilane (**a**), hydrolysis of organosilane (**b**), and schematic presentation of silica surface functionalization with organosilane (**c**).

The most commonly used modified agents to functionalize the silica surface are organosilanes, monomeric Si-containing chemicals with at least one direct silicon–carbon bond in the molecule [69]. The structure of a typical organosilane monomer consists of an organic functional group and organic moieties attached to a silicon atom (Figure 5a). During the silica surface modification reaction, the Si-OH groups react with the parent silica’s OH-groups by hydrolysis and condensation, as illustrated in Figure 5b,c. This way, the organic functional groups remain available on the surface of the silica support and can be involved in further interactions with molecules of interest.

**Figure 6:**
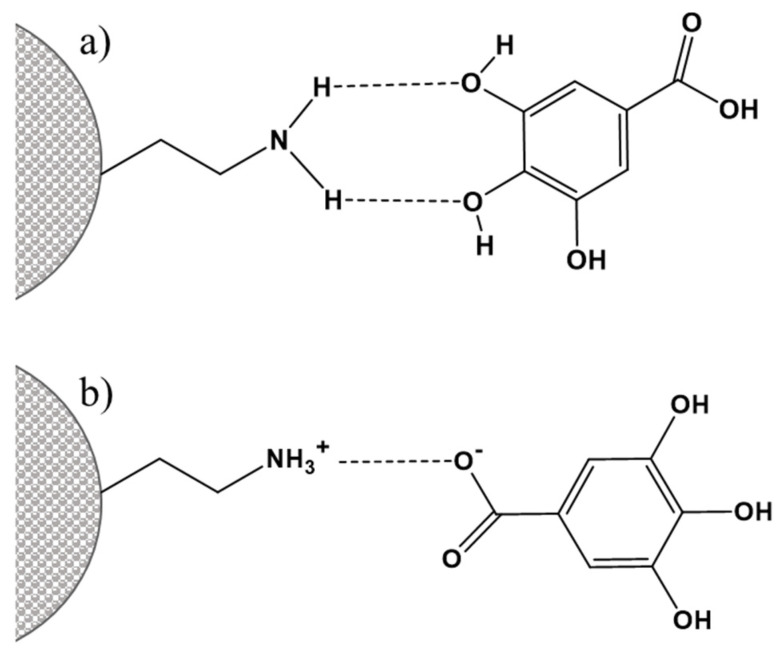
Graphical representation of hydrogen bonding (**a**) and electrostatic interactions (**b**) between adsorbent’s surface groups and the adsorbate.

The organic group in the modifying organosilane should be selected according to the reactivity of molecules for absorption/immobilization. In the cases when the reversibility of the adsorption process is desired, weaker interactions (electrostatic, H-bonds) between adsorbents and adsorbates are preferred. Figure 6 illustrates the two types of weak bonds that occur most commonly between the silica carrier and the cargo molecules.

In drug delivery systems, modification of the surface of the support could increase the affinity of the bioactive molecule to the silica support and result in more efficient loading. Furthermore, an interaction of the bioactive molecules with the appropriate group from the surface of the carrier can influence the release properties of the already adsorbed molecules, making it possible to achieve controlled release and/or targeted or triggered (photothermal, pH, enzymatic) delivery.

The surface charge of the silica carrier can be easily controlled by modifications of different organic groups. It was shown that the silica surface charge does not affect the loading of resveratrol (RES), but can play a role in the release profile of the drug at pH 7.4 and pH 5.5. Therefore, the delivery system obtained on the base of negatively charges silica (PO_3_-silica) demonstrated significantly higher anti-proliferative activity on a panel of prostate cancer PC3 cell line compared to the free RES and the system based on positively charged silica (NH_2_-silica). In the same study, it was demonstrated that functionalized silica carriers loaded with a combination of polyphenol and chemotherapeutic agent (RES and docatexal) possess improved sensitization of docatexal in hypoxia-induced drug resistance in prostate cancer [46]. 

Using silica particles modified with organic groups as carriers allows obtaining efficient antioxidant systems with a longer lifetime by preventing the deterioration of the antioxidant agent. An example of this is a system based on aminopropyl-modified silica (NH_2_-silica) with covalently immobilized gallic acid (GA). The covalent bond is realized by the formation of amide bonds between the amine groups of the silica surface and the carboxyl group of GA activated by the EDC (N-(3-dimethyl aminopropyl)-N′-ethyl carbodiimide hydrochloride) coupler. An electron paramagnetic resonance (EPR) test confirmed that the applied immobilization procedure does not compromise the antioxidant activity of the grafted GA molecules and they are capable of forming radicals and are capable of participating in radical scavenging reactions. The prepared non-antioxidant material showed excellent results in scavenging DPPH radicals via fast H-atom transfer reactions. With respect to their application, reusability tests were performed and they confirmed that the obtained materials are reusable and their RSC (radical-scavenging capacity) was not weakened after reactions with the DPPH radicals [47].

**Figure 7:**
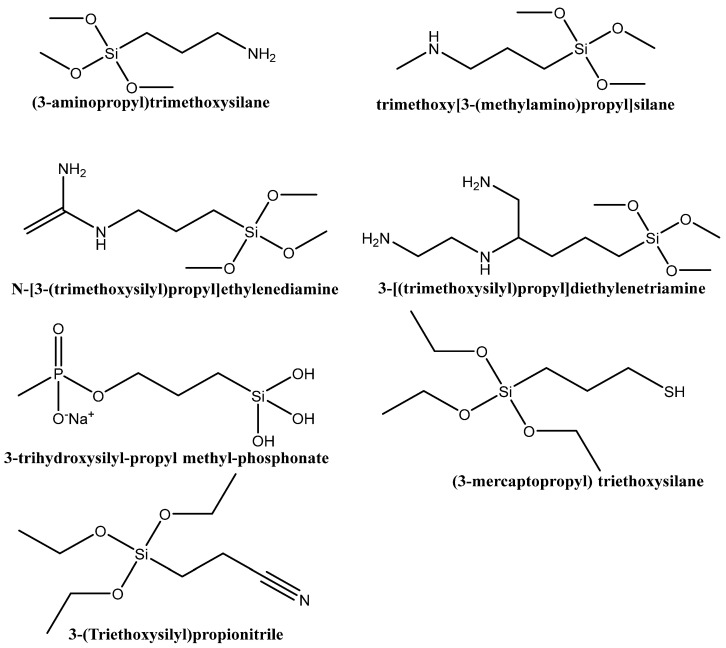
Functionalized organosilanes.

In another study, MCM-41, SBA-15, SBA-NH_2_, and SBA-SH materials were used as potential carriers of GA. It was found that the adsorption efficiencies increased in the order SBA-15, SBA-SH < MCM-41 < SBA-NH_2_. The highest absorption for amino-modified SBA-15 might be a result of ionic interactions between the positively charged silica surface amino (-NH_3_^+^) groups and the negatively charged carboxyl (R-COO^−^) group of GA. In the case of the remaining three supports, only weaker hydrogen bonds’ interactions between the hydroxyl or carboxyl groups of the GA and the silanol groups of the silica surface are possible. In the same study, the adsorption and interactions between the amino-modified surface of SBA-NH_2_ and the carboxyl group of chlorogenic acid (CGA), protocatechuic acid (PA), and 4-hydroxybenzoic acid (4-HBA) were also confirmed. The highest adsorption efficiency onto SBA-NH_2_ silica among the tested acids was noted for CGA. This tendency can be explained by the fact that the molecule of this polyphenol have the highest number of functional groups in comparison with the other three. Logically, increasing the number of hydroxyl groups in the polyphenol structure increased the absorption efficiency, as in this case it follows the order 4-HBA < PCA < GA < CGA for molecules containing 1, 2, 3, or 5 hydroxyl groups, respectively [48]. A similar phenomenon of increased adsorption for amine-modified MCM-41 in comparison with a non-modified material was observed for tannic acid (TA) as well [49]. An interesting study compares the polyphenolic acids (GA, CLA, CA, pCA, and rosmarinic acid (RA)) adsorption of mesoporous silica materials functionalized with different amino-silanes ((3-aminopropyl)trimethoxysilane, trimethoxy[3-(methylamino)propyl]silane, N-[3-(trimethoxysilyl)propyl]ethylenediamine and 3-[(trimethoxysilyl)propyl]diethylenetriamine, Figure 7). Regarding the five polyphenol compounds, the adsorbed amount of all amino-modified silicates increased in the order GA < CA < pCA < CLA < RA. The results for comparison of the absorption values for the different silica modifications showed that the carrier modified with amino-silane containing 3 nitrogen atoms had the highest adsorption of all phenolic acids, and second highest of the carrier containing 2 nitrogen atoms, and the least effective were the mono nitrogen organosilanes. It can be concluded that the increasing number of amino groups leads to increased adsorption efficiency. Аlthough there is a pattern, there are multiple interactions, such as steric hindrance, solvent effect, competitive action, hydrogen bonding, and electrostatic interactions, during the adsorption process that have to be taken into account [50]. 

The animo-modified mesoporous KIL-2 (textural mesoporosity) and order KIT-6 (interpenetrating cylindrical pore system) nanoparticles with sizes around 40 and 60 nm, respectively, were used as curcumin supports. The theoretical calculations suggest that curcumin (in both enol and keto forms) interacts weakly with the amino groups ((CH_2_)_3_NH_2_) via a phenolic single-bond OH group or via a keto group with ammonia groups ((CH_2_)_3_NH_3_^+^). The calculated vibrational frequencies are in good agreement with the obtained IR results [51].

A comparative study on parent and amino-modified MCM-41 and MCM-48 silicates as carriers of FA showed that the modification of the surface does not lead to higher loading efficiency of FA by the applied vacuum assisted technique. However, the amount loaded in MCM-41 materials exceeds the one found in MCM-48 samples. The confirmed strong interactions of the COOH group of the FA and the NH_2_ from the silica surface hinders the release on the loaded cargo. This phenomenon could be beneficial in materials for antimicrobial treatment. The antimicrobial tests confirmed that the MCM-41_modified with NH_2_ groups and loaded with FA possesses the best antimicrobial activity against Gram-positive and Gram-negative bacteria (*S. aureus*, *P. aeruginosa*, and *C. albicans*) [52].

The mesoporous silica materials modified with organic groups were also tested as supports in delivery systems for not only one type of polyphenol, but also for plant extracts containing a mixture of polyphenols. In a study, the polyphenolic extract (grape pomace) loading into MCM-41 silica functionalized with propionitrile (MCM-CN), propionic acid (MCM-COOH), mercaptopropyl (MCM-SH), and propyl sulfonic acid (MCM-SO_3_H) moieties was studied. The influence on the extract’s biocompatibility and radical scavenging activity (RSA) were evaluated for all pure silica and functionalized samples. The result showed that the stability and the RSA of the silica-embedded extract were preserved for a longer time period and the in vitro antioxidant effect was improved in comparison with that of the free polyphenolic extract. The release experiments at pH 5.7 in phosphate-buffered saline (PBS) were evidence of a relationship between the acidity of the silica surface moieties and the amount of released phytochemicals, as the main tendency is decrease of the functional groups’ acidity, leading to a decreasing of the amount released. The results from an intracellular assay showed correlation between the amount of cytosolic ROS and released polyphenolic compounds in PBS—the higher the release, the lower the ROS concentration [53].

### 6.2. Functionalization with Metal Species

**Figure 8:**
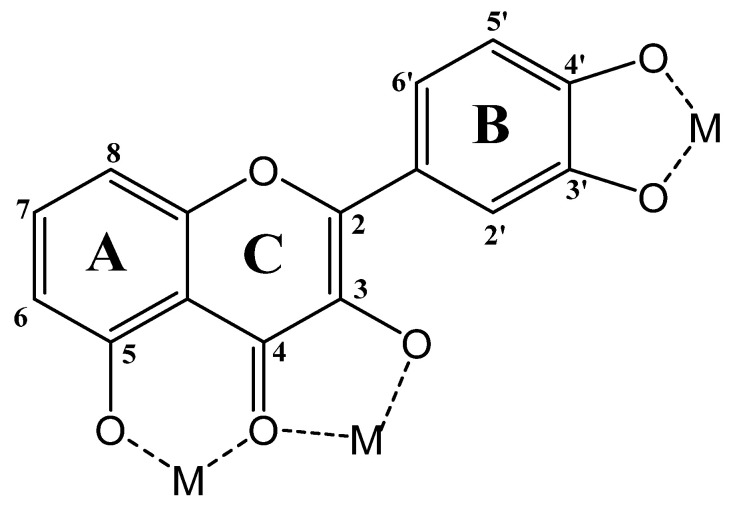
Most favorable chelating sites in flavonoid molecules.

Polyphenols are well known as a good chelating agent, due to the presence of hydroxy and keto groups in their structure [70]. The most favorable metal-chelating sites in some polyphenol molecules are: (i) the 3-hydroxy-4-ketone groups in the C-ring, (ii) the 5-hydroxy group in the A-ring and 4-carbonyl group in the C–ring, and (iii) 3′,4′-dihydroxy groups in the B-ring (Figure 8) [71]. 

Studies have shown that the formation of polyphenol–metal ion complexes can affect the properties of the parent biologically active molecules and lead, in most cases, to superior antioxidant, antimicrobial, anticancer, and antidiabetic activities, significantly increasing their therapeutic potential [72,73]. For example, the coordination of kaempferol in complexes Ca(II), Sr(II), and Ba(II), or coordination of quercetin, rutin, galangin, and catechin with Cu(II), Fe(II), Al(III), and Zn(II) ions increases their radical scavenging efficiency, as well as their antiproliferative activities on various human cancer cell lines [74,75,76,77]. In an in vivo experiment, a vanadium complex of kaempferol was found to possess better antihyperglycemic activity compared to the free flavonoid [78]. Published data demonstrate that the oxidovanadium(IV) complexes with baicalin, apigenin, silibinin, and luteolin have superior antimetastatic action on a human lung cancer cell line in comparison with the free ligands [79]. Several studies suggested synergistic effects of polyphenolic ligands in complex with Ru(II)/(III) and, in the majority of the cases, the obtained compounds showed a greater improvement in antiproliferative and/or enzyme inhibitory activity than was observed for the polyphenols themselves [80]. The metal complexation of flavonoids results in better pharmacological activities and the complexes are characterized with higher stability in vitro, like in vivo conditions [81]. 

**Figure 9:**
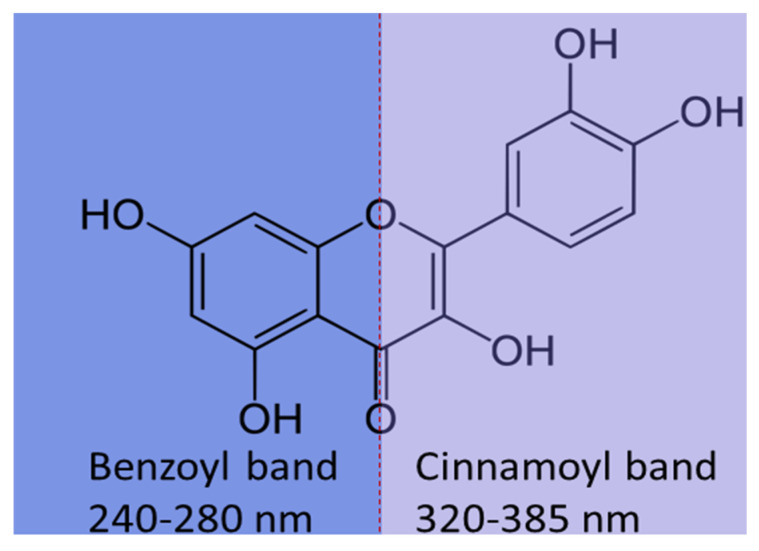
UV–vis absorption regions for benzoyl and cinnamoyl structures (quercetin molecule is used as an example).

The formation of these complexes also changes the physicochemical properties of the molecules, such as their absorbance in the UV–vis region, which makes UV–vis spectroscopy a suitable method for the detection of complexation. Due to electronic π–π* transitions, flavonoids are characterized with two absorption bands in the UV–vis region, benzoyl and cinnamoyl at 240–280 nm and 320–385 nm, respectively (Figure 9), which are bathochromically shifted after chelating with metals [73,82].

Nevertheless, some of the major drawbacks of these compounds, such as hydrophobicity and low solubility, cannot be overcome just by simple complexation with metal ions. On the other hand, polyphenols’ ability to interact with metal ions is a promising approach to achieving higher loading capacity by modifying suitable drug carriers with an appropriate metal. Over the past decade, published data suggest metal-modified mesoporous silica materials with different pore structure, particles size, and shape as favorable supports for biologically active polyphenols. Usually, for biomedical applications, silica surface-modifying species are used with metal nanoparticles/ions that have beneficial health properties, such as Ca, Ag, Zn, Mg, Fe [83,84,85,86,87,88]. This way, they can act not only as an attaching moiety for the bioactive molecules, but also contribute to the prevention/treatment of the target problem. 

**Figure 10:**
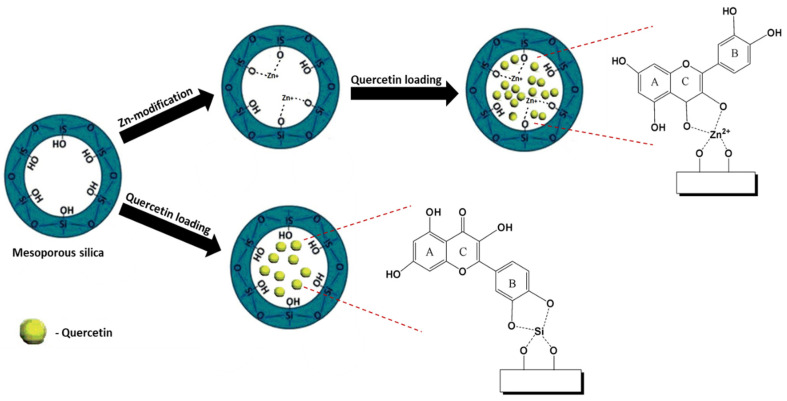
Schematic representation of the quercetin interactions with the surface OH groups from the non-modified and the Zn cation from the modified silica carriers.

For example, quercetin was loaded on pure silica types MCM-41, SBA-15, and SBA-16, and on Zn-modified analogues prepared by a post-synthesis method (Figure 10). Spectroscopic data suggest interactions between quercetin and the surface of pure or Zn-modified mesoporous silicates, confirming the formation of a Zn-quercetin complex. Theoretical calculations confirmed quercetin had a higher binding affinity for the Zn^2+^ cation than the silanol groups, which were the only functional groups present on the surface of the parent silica. The higher affinity of the quercetin to Zn-containing supports leads to a slower in vitro release process at pH 5.5 PBS, in comparison with formulations based on non-modified silica carries. The comparative cytotoxic experiments for the formulations on the basis of SBA-15 mesoporous support confirmed the superior antineoplastic potential against HUT-29 cells of the Q immobilized on Zn-modified supports compared to that of the free drug [54,55].

Another system, based on Ag-modified MCM-41 porous silica, for topical administration of quercetin has been proposed, in which the health-beneficial properties of this polyphenol were combined with those of the silver. For the incorporation of the Ag-species in the silica matrix, two different approaches were applied: (1) modification by direct synthesis; or (2) post-synthesis methods. The in vitro release process at a pH suitable for dermal formulations (5.5) showed lower and incomplete quercetin release for silver-modified samples in comparison with the parent MCM-41. A possible explanation of the observed results is the formation of a strong complex between quercetin and Ag. The high quercetin loading (over 40%) and slower release indicates that the obtained delivery systems are promising for dermal application. The cytotoxicity experiments show that Ag-modified and quercetin-loaded silica carriers prepared by the post-synthesis method exert superior antineoplastic potential against HUT-29 cells compared to free drugs [56].

In another study, systems for the delivery of morin and hesperetin were designed on the basis of Ag- and Mg-containing SBA-16 nanoparticles. The post-synthesis procedure for modification led to the incorporation of Mg in the silica framework as ionic species, while for the silver, the formation of nanoparticles present in the channels of the carrier and on the outer surface of silica particles was observed. In vitro experiments reveal that the formation of metal-flavonoid complexes influences the release of the loaded molecules. It was found that the morin release depends on available surface groups because of the different affinity of its molecules to the surface moieties (-OH, Ag, Mg), while for hesperetin the effect of the carrier surface modification does not affect its release properties, most probably due to the less pronounced interaction of the drug and the carriers. The evaluation of the cytotoxicity and the antioxidant capacity of the obtained delivery systems showed improved properties in comparison with the pure morin and hesperetin [57]. Another work on the topic of Mg-containing silica (MCM-41) studied the influence of the modification procedure (in situ, template ion exchange, incipient wetness impregnation) on the physicochemical and pharmacokinetic properties of the obtained delivery system based on these carriers. It was demonstrated that the efficiency of the Mg incorporation and materials’ textural properties strongly depended on the applied approach. The as-prepared materials were studied as carriers for kaempferol in a delivery system for oral administration. The loading of the flavonoid into the Mg-silica supports led to improvement of kaempferol’s solubility. It was confirmed that the RSA against DPPH radicals of the flavonoid loaded into the parent and post-synthesis Mg-modified silica supports was not compromised during the process of encapsulation. In contrast, for the delivery system on the basis of MCM-41 modified with Mg by direct synthesis, the RSA is decreased nearly by half, which could be the effect of the formation of a strong Mg–kaempferol complex with the Mg species incorporated into the silica walls during the formation of the material. In conclusion, different approaches for silica modification with Mg can be used to obtain materials with desired properties [58].

**Figure 11:**
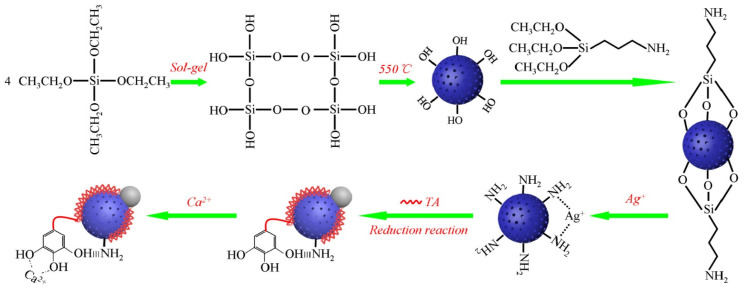
Step-by-step diagram of the synthesis of Ca-TA-MSN@Ag. Reproduced here with permission from [59], Elsevier, 2021.

On the basis of Ag-containing silica particles, a system for faster healing of wounds by achieving rapid hemostasis and preventing bacterial infection has been developed. A complex system with a Janus structure containing mesoporous silica nanoparticles decorated by tannic acid, silver nanoparticles, and calcium ions was obtained in a stepwise manner by reactions of surface modification with NH_2_-groups, Ag coordination, followed by a reduction reaction between tannic acid and Ag^+^, and finally Ca^2+^ coordination (Ca-TA-MSN@Ag, Figure 11). The as-obtained material accelerated the coagulation reaction and caused faster fibrin network formation. These formulations showed excellent biocompatibility and antibacterial activity (~99%) against *E. coli* and *S. aureus* [59].

**Figure 12:**
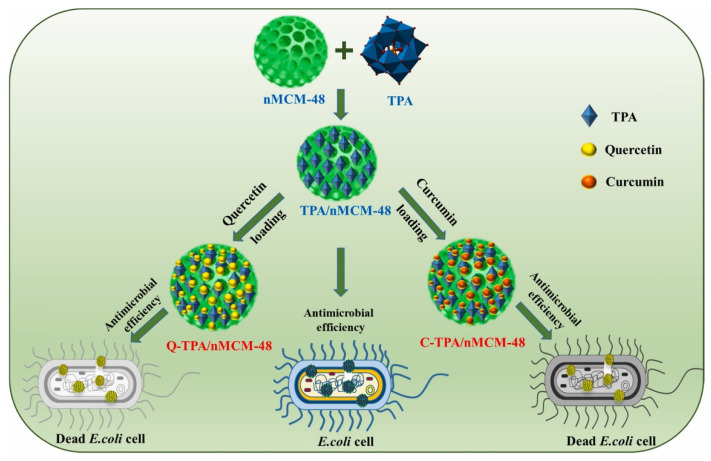
Schematic representation of the synthesis strategy and antimicrobial action of Q-TPA/MCM-48 and C-TPA/MCM-48. Reproduced here with permission from [60], Elsevier, 2023.

In a recently published work, phosphotungstic acid (TPA) was used for functionalization of MCM-48 nanoparticles’ surface and the obtained material was explored as a carrier in delivery systems for curcumin (CUR) and quercetin (Q). Phosphotungstic acid was chosen because of its potential for obtaining conjugated nanomatrices for highly effective and selective medical applications. The release profiles of Q and CUR loaded in modified MCM-48 were evaluated in PBS at different pH values (5, 6.2, 7.4). C- and Q- loaded TPA/MCM-48 nanoparticles demonstrated prolonged and sustainable drug release for 60 h, and exhibited significant antibacterial activity against *E Coli.* Figure 12 is a schematic representation of the synthesis strategy and antimicrobial action of Q-TPA/MCM-48 and C-TPA/MCM-48. The loaded amount of both polyphenols into the modified porous silica was very close (86 mg for CUR and 84 mg for Q in 100 mg nanocomposite). These results show that neither of the bioactive molecules had stronger affinity to the modifying surface groups. On the other hand, faster release and slightly higher burst release of CUR in comparison with that of Q, where also the pH of the release medium played a role in the speed and the amount of release, was observed. As the optimal condition, pH 7.4 was chosen. For both substances, sustainable and prolonged release (up to 60 h) from the phosphotungstic-modified silica was achieved. The significant antimicrobial activity of the modified silica and modified silica loaded with CUR or Q against *E. coli* was proven, and the loaded formulations exhibited twice the activity of the modified support alone [60].

Calcium silicate-based composites also attract significant attention from researchers due to their excellent performance in bone tissue regeneration treatments. To increase their biocompatibility, antimicrobial activity, and anti-inflammatory effect these materials are successfully combined with natural polyphenols such as gallic acid (GA), pyrogallol (PG), and tannic acid (TA). Published data reported the influence of the polyphenols’ concentration on the setting time, antibacterial activity against *E. coli* and *S. aureus*, and osteogenic activity on human osteoblast-like cell line MG63 of the obtained samples. The results showed that the loading of polyphenols in calcium silicate greatly enhanced its antibacterial activity, and did not have a significant effect on the osteogenic activity (MG63 cells) and the cytotoxicity (L929 cells) [61]. It was found that quercetin-containing mesoporous calcium silicate carriers obtained by polycaprolactone-assisted 3D printing process possess great bioactivity and mechanical properties for the promotion of osteogenesis in mesenchymal stem cells. The mesoporous calcium silicate scaffold loaded with quercetin exhibited greater results in in vitro tests for cell proliferation, cytotoxicity, and immunofluorescence staining for mesenchymal stem cells in comparison with the non-loaded composites. This novel approach for the preparation of efficient materials for bone tissue regeneration at room temperature could be used instead of bone-related proteins, which remain bioactive only at low temperatures [62]. In another study, genistein (GN) was used to enhance the cells’ (MC3T3-E1) response (adhesion, proliferation, differentiation, and gene expressions) in vitro and to promote osteogenesis in vivo of mesoporous magnesium-calcium-silicate/polyetheretherketone composite with potential application in bone regeneration. The obtained composite contains 40 wt% mesoporous magnesium-calcium-silicate loaded with genistein, and it was prepared by a cold pressing and sintering method. Based on a comparative study, it can be concluded that the presence of genistein stimulated the cell responses in vitro and significantly improved osteogenesis and enhanced osseointegration of the parent composite [63].

Silica materials modified with metal oxides were used as carriers not only for one isolated molecule but for polyphenolic extracts as well. The stability of extracts from grape pomace was enhanced by their encapsulation in pure mesoporous silica type MCM-41 and decorated with ZnO (Zn-MCM-41) or MgO (Mg-MCM-41) analogues. The stability of free and encapsulated extracts was studied for evaluation of their radical scavenger activity in time, assessed by DPPH method. The encapsulated extracts demonstrated similar antioxidant capacity up to 5 months, whereas the free extracts showed a decrease of their activity over time due to the degradation. The best cytocompatibility was obtained for Zn-MCM-41 encapsulated extract, which makes it a promising candidate for incorporation in cosmetic or nutraceutical formulations [64].

Additionally, NH_2_-modified silica could be applied as a layer around magnetic iron oxide nanoparticles as a core in order to obtain inorganic composites (average particle size 50 nm) with application as adsorbents for flavonoids extracted from licorice (*Glycyrrhiza uralensis Fisch*.) root. A comparative study showed greater affinity and faster attainment of the adsorption equilibrium of the flavonoids to the silica-iron oxide particles than to the commercial absorbents. The higher purity of the enriched extract and the easy desorption of flavonoids from these adsorbents make them promising in magnetic separation technology for natural products [65]. 

In a few recent studies, different approaches for using polyphenol–metal complexes in combination with porous silica in controlled drug delivery were demonstrated. The polyphenols’ ability to form complexes with metal ions is used to form trigger-sensitive coatings around the silica particles. These new types of composite materials possess excellent stability and biocompatibility in a physiological environment. The high specific surface area of silica-porous nanoparticles allows the loading of a significant amount of biologically active molecules, while the coating of metal–polyphenolic networks assures the photothermal and pH-responsive properties. The nature of these carriers makes them promising candidates for applications in photothermal and pH-sensitive therapy.

In these cases, the formation of a complex of tannic acid (TA) with metal ions (Fe^3+^, Al^3+^) was applied in order to encapsulate drug-loaded silica particles. The preparation of such systems usually requires the following steps: i) synthesis of the silica carrier; ii) loading the bioactive substance in the pores of the carrier; iii) encapsulation of the loaded particles in a polyphenol–metal framework by self-assembly process. Results for the delivery system of fucoxanthin (natural carotenoid) based on Fe_3_O_4_-SiO_2_-TA nanoparticles obtained by the above-described procedure offer magnetic and pH-dependent targeted delivery. Improved water dispersion and biocompatibility, as well as inhibition of growth of human colon cancer cells (HCT116) and low cytotoxicity against mouse fibroblast cells (L929) were shown from the obtained formulations compared to free fucoxanthin [66]. The same strategy for encapsulation of the anti-tumor drug doxorubicin resulted in an improvement in the effectiveness of the treatment and superior biocompatibility [89]. In these systems, by controlling the thickness of the coating of the polyphenol–metal network, the photothermal performance of the obtained delivery system can be easily tuned, which makes these materials promising candidates not only for pH-dependent therapy but also for photothermal therapy [90]. Another example for a similar system was designed on the basis of polyacrylic acid-coordinated Mn^2+^ and F^−^ co-doped nanoscale hydroxyapatite coated with metal–polyphenol network. The coating of pH-sensitive tannic acid (TA)-Fe^3+^ complex improves the biocompatibility of the delivery system, increases the stability of the hydroxyapatite carrier, preventing the burst release of the loaded drug (doxorubicin) before reaching the target (tumors), and greatly enhanced the drug loading and encapsulation efficiency. After phagocytosis by HeLa cells, the obtained delivery system degrades rapidly while continuously releasing the loaded antitumor drug (doxorubicin), TA, and Mn^2+^ ions. The released Mn^2+^ ions have the ability to bind to proteins, which leads to enhanced magnetic resonance contrast. The developed pH-sensitive and magnetic resonance imaging-active delivery system showed great potential for tumor diagnosis and therapeutic synergy [67].

## 7. Conclusions and Final Remarks

Recent advancements in the development of novel tools to enhance quality of life are driving research towards engineering of highly effective materials for diagnostic and therapeutic applications. The unique properties of mesoporous silica materials make them ideal for designing delivery systems of biologically active compounds. Used as carriers mesoporous silica, they can be a solution to some serious drawbacks of bioactive molecules, like polyphenols, such as low solubility, burst release and fast metabolism before the target organ/tissue is reached, inability to internalize the target cells, fast degradation, etc. Rapid progress in developing new porous silica has resulted in materials with diverse particle sizes, shapes, pore structures, and surface properties. Comparative studies were conducted to identify the most suitable carriers for specific biologically active compounds. The data indicate that pore structure and size significantly influence drug loading effectiveness and can affect the kinetics of drug release. These parameters are determined by steric and diffusion factors, including the size of the adsorbate molecules, their ability to form crystals, and their solubility. Both the loading capacity and release behavior are also greatly influenced by the surface chemistry of the porous silica carrier and the loaded molecules. The easy-to-modify silica surface allows enhancing the affinity of the adsorbate molecules to the carrier by introducing specific selective species, such as organic groups or metal ions. By increasing the potential for ionic, electrostatic, or hydrogen bond interactions, the loading efficiency and release properties of the delivery system can be improved. The results indicate that an increased number of specific surface groups leads to higher adsorption efficiency. However, due to the complexity of creating an effective delivery system, no strict rules can be established for the optimal pore structure or surface modification.

## Figures and Tables

**Table 1:** The application of different porous materials for polyphenol encapsulation: structural characteristics, surface modification groups, and loading capacity.

Type of Silica	Pore Structure	Average Pore Size, nm	Surface Modification	Loaded Polyphenol (Amount)	Ref.
MCM-41	2D cylindrical mesopores, hexagonal arrangement	2.7	-	RES (32 wt%)	[40]
KIL-2	Wormhole-like	15.2	-	RES (37 wt%)	[40]
Zeolite BEA	3D	-	-	RES (40 wt%)	[40]
MCM-41	2D cylindrical mesopores, hexagonal arrangement	-	-	CA (45 wt%), p-CA (39 wt%), FA (33 wt%)	[41]
MCM-48	3D interlaced pore system divided by a continuous pore wall	--	-	CA (35 wt%), p-CA (34 wt%), FA (32 wt%)	[41]
HMS	Wormhole-like	2.5	-	Q (40% EE **), NAR (41% EE)	[42]
MSU-2	Wormhole-like	3.1	-	Q (40% EE), NAR (43% EE)	[42]
SBA-15	2D hexagonal	5.6	-	Q (32% EE), NAR (47% EE)	[42]
SBA-16	3D cage-like, body-centered-cubic array	3.3	-	G	[43]
KIT-6	3D interpenetrating cylindrical, bicontinuous cubic	5.7	-	GA	[43]
MCM-41	2D cylindrical mesopores, hexagonal arrangement	3.1	-	GA	[43]
ULPFDU-12	3D spherical cage-like, face-centered cubic	4.7	-	GA	[43]
Q10 SiO_2_ ***	1D	18.6	-	GA	[43]
MSU-F	cellular foam	16.4	-	GA	[43]
MSN *	Wormhole-like	1.8	NH_2_	RES (10 wt%)	[46]
MSN *	Wormhole-like	2.3	PO_3_	RES (11 wt%)	[46]
Aerosil A90 ***	Solid particles	-	NH_2_	GA (100 μM/g)	[47]
Aerosil A150 ***	Solid particles	-	NH_2_	GA (152 μM/g)	[47]
Aerosil A300 ***	Solid particles	-	NH_2_	GA (259 μM/g)	[47]
Aerosil A380 ***	Solid particles	-	NH_2_	GA (366 μM/g)	[47]
MCM-41	2D cylindrical mesopores, hexagonal arrangement	2.7	-	GA (19%)	[48]
SBA-15	2D hexagonal	6.3	-	GA (13% EE)	[48]
SBA-15	2D hexagonal	6.3	NH_2_	GA (26% EE)	[48]
SBA-15	2D hexagonal	6.3	SH	GA (13% EE)	[48]
SBA-15	2D hexagonal	6.3	NH_2_	CGA (55% EE)	[48]
SBA-15	2D hexagonal	6.3	NH_2_	PA (8% EE)	[48]
SBA-15	2D hexagonal	6.3	NH_2_	4-HBA (3% EE)	[48]
MCM-41	2D cylindrical mesopores, hexagonal arrangement	2.4		TA	[49]
MCM-41	2D cylindrical mesopores, hexagonal arrangement	2.4	NH_2_	TA	[49]
MSN	-	8.4	NH_2_	GA (36 μmol), CLA (48 μmol), CA (40 μmol), pCA (46 μmol), RA (53 μmol)	[50]
MSN	-	8.8	NHCH_3_	GA (30 μmol), CLA (46 μmol), CA (31 μmol), pCA (46 μmol), RA (58 μmol)	[50]
MSN	-	8.8	NHC_2_H_4_NH_2_	GA (48 μmol), CLA (60 μmol), CA (48 μmol), pCA (60 μmol), RA (76 μmol)	[50]
MSN	-	8.6	NHC_2_H_4_NHC_2_H_4_NH_2_	GA (60 μmol), CLA (70 μmol), CA (68 μmol), pCA (68 μmol), RA (83 μmol)	[50]
KIL-2	Wormhole-like	12.5	NH_2_	CUR (28 wt%)	[51]
KIT-6	3D interpenetrating cylindrical, bicontinuous cubic	5.5	NH_2_	CUR (28 wt%)	[51]
MCM-41	2D cylindrical mesopores, hexagonal arrangement	-	-	FA (33 wt%)	[52]
MCM-41	2D cylindrical mesopores, hexagonal arrangement	-	NH_2_	FA (32 wt%)	[52]
MCM-48	3D interlaced pore system divided by a continuous pore wall	-	-	FA (32 wt%)	[52]
MCM-48	3D interlaced pore system divided by a continuous pore wall	-	NH_2_	FA (26 wt%)	[52]
MCM-41	2D cylindrical mesopores, hexagonal arrangement	3.9	-	grape pomace extract	[53]
MCM-41	2D cylindrical mesopores, hexagonal arrangement	3.2	CN	grape pomace extract	[53]
MCM-41	2D cylindrical mesopores, hexagonal arrangement	3.2	COOH	grape pomace extract	[53]
MCM-41	2D cylindrical mesopores, hexagonal arrangement	3.5	SH	grape pomace extract	[53]
MCM-41	2D cylindrical mesopores, hexagonal arrangement	3.7	SO_3_H	grape pomace extract	[53]
MCM-41	2D cylindrical mesopores, hexagonal arrangement	2.7	-	Q (35 wt%)	[54]
MCM-41	2D cylindrical mesopores, hexagonal arrangement	2.7	Zn (2/4%)	Q (42/37 wt%)	[54]
SBA-16	3D cage-like, body-centered-cubic array	3.6	-	Q (41 wt%)	[54]
SBA-16	3D cage-like, body-centered-cubic array	4.6	Zn (2/4%)	Q (43/45 wt%)	[54]
SBA-15	2D hexagonal	6	-	Q (42 wt%)	[55]
SBA-15	2D hexagonal	5.8	Zn (2/4%)	Q (44/46 wt%)	[55]
MCM-41	2D cylindrical mesopores, hexagonal arrangement	4.2	Ag	Q (44 wt%)	[56]
SBA-16	3D cage-like, body-centered-cubic array	4.5	-	MOR (29 wt%), HES (30 wt%)	[57]
SBA-16	3D cage-like, body-centered-cubic array	4.4	Ag	MOR (33 wt%), HES (32 wt%)	[57]
SBA-16	3D cage-like, body-centered-cubic array	4.4	Mg	MOR (32 wt%), HES (28 wt%)	[57]
MCM-41	2D cylindrical mesopores, hexagonal arrangement	2.2	-	KM (25 wt%)	[58]
MCM-41	2D cylindrical mesopores, hexagonal arrangement	2.7	Mg	KM (30 wt%)	[58]
MSN	-	3.7	NH_2_/Ag/TA/Ca^2+^	-	[59]
MCM-48	3D interlaced pore system divided by a continuous pore wall	2.25	TPA	CUR (84 wt%), Q (86 wt%)	[60]
Calcium-silicate composite	-	-	-	GA, PG, TA	[61]
Mesoporous calcium silicate-calcium sulfate (MSCS)/polycaprolactone (PCL)	-	-	-	Q	[62]
Mesoporous magnesium-calcium-silicate/polyetheretherketone composite	-	-	-	GN	[63]
MCM-41	2D cylindrical mesopores, hexagonal arrangement	-	-	grape pomace extract (48 wt%)	[64]
MCM-41	2D cylindrical mesopores, hexagonal arrangement	-	ZnO	grape pomace extract (50 wt%)	[64]
MCM-41	2D cylindrical mesopores, hexagonal arrangement	-	MgO	grape pomace extract (47 wt%)	[64]
Magnetic silica particles	-	-	NH_2_	licorice (*Glycyrrhiza uralensis Fisch*.) root extract	[65]
Magnetic silica particles	-	3.4	Fe^3+^, Al^3+^	TA	[66]
Polyacrylic acid-coordinated Mn^2+^ and F^−^ co-doped hydroxyapatite	-	6	Fe^3^	TA	[67]

MSN * mesoporous silica nanoparticles; EE ** encapsulation efficiency; *** commercially available materials.

## Data Availability

The review summarized published results and no new data were created.
